# The Mutation of myomiR miR499 Impacts the Intermuscular Bones in Zebrafish

**DOI:** 10.3390/biology14121670

**Published:** 2025-11-25

**Authors:** Jinyuan Che, Yidong Feng, Haichuan Li, Qi Wang, Chunxin Fan, Baolong Bao

**Affiliations:** Key Laboratory of Exploration and Utilization of Aquatic Genetic Resources, Ministry of Education, National Demonstration Center for Experimental Fisheries Science Education, Shanghai Ocean University, Shanghai 201306, China; fyd0531@126.com (Y.F.); 15380686273@163.com (H.L.); 13752109175@163.com (Q.W.); cxfan@shou.edu.cn (C.F.)

**Keywords:** intermuscular bones, miR499, muscle, *sox6*, tail-beat frequency

## Abstract

The development of intermuscular bones (IBs) in teleost fish is regulated by osteogenic genes, but the upstream signals that initiate ossification remain elusive. Given the spatiotemporal correlation between IB formation and swimming behavior, mechanical stimuli from muscle activity have been hypothesized as potential regulators. In this study, we investigated the role of the muscle-specific microRNA miR499 in this process. Using CRISPR/Cas9-generated miR499 knockout zebrafish, we demonstrated that the mutant of miR499 induces a fast-twitch muscle phenotype through the upregulation of *sox6*, accompanied by increased fast-myosin gene expression and fast-twitch fiber proliferation. This shift led to distinct locomotor alterations, characterized by reduced tail-beat frequency and increased stride length. Crucially, these changes resulted in delayed IB ossification onset, slower ossification rates, and simplified morphology. The total ossification area decreased by 27%. Molecular analysis revealed a downregulation of osteogenic genes in IB tissue. Our findings establish miR499 as a key regulator of a muscle–bone functional axis, where miRNA-mediated muscle fiber specification determines mechanical output that subsequently guides IB development. This work provides new insights into the mechanobiological regulation of skeletal development and identifies a promising genetic target for aquaculture breeding programs aimed at attenuated IBs.

## Simple Summary

Why do some fish have many intermuscular bones (IBs) within their muscle? Traditional research has focused on genes directly involved in bone formation to answer this question. In this study, we investigated the problem from a new perspective: the role of muscle itself. Using gene-editing technology, we created zebrafish with mutant miR499, a small molecule active only in muscle tissue. We found that this muscle-specific alteration not only changed the fish’s swimming pattern but also significantly delayed and reduced the development of intermuscular bones. Our work provides direct evidence that genetic signals in muscle tissue serve as crucial upstream regulators commanding IB development. This discovery shifts the research focus from bone to muscle and suggests new possibilities for breeding fish with reduced intermuscular bones by targeting muscle-specific genes.

## 1. Introduction

Intermuscular bones (IBs) are a unique type of dermal bone found specifically in teleost fishes, widely distributed among groups such as Cyprinidae and Anguillidae [1,2,3]. They play a critical role in maintaining mechanical stability in fish and coordinating muscle contraction to support different swimming modes [3]. Furthermore, IBs are closely associated with evolutionary adaptation and economically important traits in aquaculture. For instance, the presence of IBs directly influences the processing quality and eating convenience of fish flesh. Therefore, elucidating the mechanisms underlying their formation holds significant theoretical importance for understanding the evolution of intramembranous ossification in teleosts, as well as practical value for breeding improved aquaculture varieties lacking IBs [4,5]. Consequently, understanding the molecular mechanisms governing IB development is of both fundamental biological interest and significant applied importance.

Previous studies have examined variations in the number and morphology of IBs across different farmed fish populations, with preliminary quantitative trait locus (QTL) mapping identifying genomic regions associated with IB number in common carp [2,5,6,7,8,9,10,11]. Further investigations have characterized gene expression profiles within the myosepta during fish development [12], while transcriptomic and proteomic analyses have uncovered molecular signatures of IB formation [13,14]. Gene editing approaches have firmly established *runx2b* and *bmp6* as core regulators of IB development, both playing pivotal roles in osteoblast differentiation [15,16]. Specifically, Runx2b drives the differentiation of tendon progenitor cells in the myosepta into osteoblasts via regulating osteogenic marker genes such as *alpl*, *osterix*, and *spp1*, while the targeted knockout of *bmp6* eliminates IB formation entirely in cyprinid fish without impairing viability [15,16,17].

These factors initiate the intramembranous ossification characteristic of IBs-a process sharing fundamental mechanisms with other skeletal elements: the Runx2b-mediated regulation of bone matrix genes and BMP family signaling are conserved across vertebrate skeletal development [18,19], and TGF-β/BMP pathway enrichment is observed in both IB and non-IB osteogenic processes. A particularly intriguing evolutionary puzzle emerges: why do some teleosts possess IBs while others do not, despite the universal presence of *runx2b* and *bmp6*? *Runx2b* is conserved in IB-bearing species (e.g., zebrafish, blunt snout bream) and fish without IBs (e.g., medaka) [16,20,21]. Similarly, *bmp6* homologs exist in IB-absent taxa such as Nile Tilapia [14,17,22,23,24]. Thus, the presence or absence of IBs is not determined by the mere existence of *runx2b* and *bmp6* but by the upstream signaling pathways and genetic networks that modulate their expression and activity in the myoseptal microenvironment.

Studies have established that IBs develop through a typical process of intramembranous ossification: originating from mesenchymal progenitor cells within the myosepta, they differentiate directly into osteoblasts and deposit bone matrix without undergoing a cartilaginous stage [25,26,27,28]. It is noteworthy that the ossification of IBs follows a distinct spatiotemporal sequence, which is strongly correlated with the swimming mode of the fish [29,30]. In subcarangiform or carangiform swimmers (such as *Hypophthalmichthys molitrix* and *Megalobrama amblycephala*), propulsion relies mainly on powerful oscillations of the posterior body section; the ossification of IBs in these species consistently proceeds in a posterior-to-anterior direction [31,32,33]. In *Hypophthalmichthys molitrix*, for example, IBs first appear in the caudal myosepta 40–52 days post-hatching and gradually extend toward the anterior regions [15,27]. This shared feature suggests that the posterior-driven swimming mode may have evolutionarily selected for a corresponding ossification strategy. In contrast, anguilliform swimmers propel themselves through whole-body undulatory movements, and their IBs follow a reverse anterior-to-posterior ossification sequence. Our earlier research on *Anguilla japonica* first revealed that IBs initially form in the anterior myosepta before progressively extending toward the tail [34]. This finding has been independently confirmed in *M. anguillicaudatus* by Nie et al. [30], further supporting the notion of a relationship between swimming mode and ossification sequence. These observed differences provide key phenotypic evidence supporting the biomechanical hypothesis proposed by Danos & Staab [35] that mechanical forces generated by muscle contractions during swimming may directly induce the initiation and directional spread of IB ossification [34,36]. Notably, *actn3b*, a gene involved in muscle contraction regulation, is significantly enriched in tambaqui (*Colossoma macropomum*, an IB-lacking species), indicating that muscle tension may be an important signal-related factor regulating IB genesis [37].

The undulatory swimming of most fish is powered by the lateral muscle, a structure composed of serial myomeres demarcated by myosepta. Coordinated contractions of these myomeres cause the body and caudal fin to bend. The compelling spatiotemporal correlation between IB morphogenesis and the onset of forceful muscular activity points to a model in which mechanical forces and/or myokine signals from contracting muscle play a key role in inducing or modulating the osteogenic program in adjacent connective tissue. It is hypothesized, therefore, that genes fundamental to muscle development, function, or activity are upstream regulators of the formation of these interconnected musculoskeletal structures [38]. To directly test this hypothesis—that muscle-derived signals regulate IB development—a targeted genetic approach is required.

miR499, a member of the myomiR family, exhibits restricted tissue specificity, with predominant expression in skeletal muscle (especially slow-twitch fibers) and cardiac muscle from embryonic development through adulthood [39]. In teleosts including zebrafish, medaka, and Nile tilapia, miR499 expression is tightly linked to slow muscle fiber identity—its transcript levels are up to 44-fold higher in red skeletal muscle than in white muscle, where it directly represses targets like *sox6* to maintain slow-twitch fiber phenotypes [39,40,41]. Even in lineages where its ancestral host gene *myh7ba* has been lost (e.g., medaka), miR499 retains conserved cis-regulatory elements that drive muscle-specific expression, underscoring the evolutionary constraint on its tissue-specificity. This strict expression pattern stands in stark contrast to ubiquitous muscle regulators (e.g., MyoD family genes), whose perturbation often affects multiple tissue lineages due to broader developmental roles. miR499 is an ideal molecular tool for this investigation due to its exclusive and robust expression restricted to skeletal muscle tissues. It plays well-documented, conserved roles in promoting myogenesis, maintaining muscle fiber type identity, and regulating muscle function [40,42,43]. Crucially, its strict muscle-specific expression profile ensures that genetic knockout is unlikely to exert direct effects on chondrogenesis, osteogenesis occurring outside the muscle connective tissue environment, or other non-muscle organ systems. This specificity allows for the isolation of muscle-derived effects on IB formation.

Accordingly, in the present study, we established a miR499 knockout model in a relevant teleost species and conducted phenotypic analyses, aiming to determine whether a perturbation of this key muscle-specific regulatory gene affects the initiation, patterning, and abundance of IBs. This targeted approach offers a robust method to elucidate the regulatory role of muscle-derived genetic signals—and potentially mechanical signals—in the complex process of IB formation.

## 2. Materials and Methods

### 2.1. Zebrafish Husbandry and Generation of miR499 Mutant Lines

Wild-type (WT) zebrafish (AB strain) were maintained under standard laboratory conditions as previously described [44]. The fish were maintained at 28.5 °C on a 14:10 h (light:dark) cycle. The miR499 knockout line was generated using the CRISPR/Cas9 system. Two single-guide RNAs (sgRNAs) were designed using the UCSC Genome Browser online tool. The sgRNA sequences were as follows: Target 1 of sgRNA (sgRNA1): taatacgactcactata-GGACTTTGTAGACTCAGAACgttttagagctagaa; Target 2 of sgRNA (sgRNA1): taatacgactcactata-GGCTGCCTCCCTCTCAGTATgttttagagctagaa). Transcription templates were generated by PCR using T7-targetsite-F primers and a universal reverse primer. The PCR products were purified using the Zymo RNA Clean & Concentrator-5 kit (R1015, Zymo Research, USA). Cas9 mRNA was synthesized from the linearized pT3TS-nCas9n plasmid using the Ambion mMESSAGE mMACHINE T3 Transcription Kit (AM1348, ThermoFisher Scientific, Waltham, MA, USA). A mixture of Cas9 mRNA and sgRNAs was microinjected into one-cell stage zebrafish embryos. Mutants were identified using CRISPR-STAT way by capillary electrophoresis (CE) and confirmed by sequencing.

The injected embryos were placed in E3 medium (5 mM NaCl, 0.17 mM KCl, 0.33 mM CaCl_2_, 0.33 mM MgSO_4_) in a 28.5 °C constant temperature incubator. At 3 dpf, DNA from 10 larvae was extracted, the region containing the target site was amplifed via PCR, and the miR499 deletion was confirmed by DNA sequencing. Primary embryos (F_0_) were raised to adulthood and mated with WT zebrafsh to obtain F1 generation embryos, which were raised to adulthood. The tails of F1 adult zebrafsh were collected, and the DNA was extracted, amplifed via PCR, and sequenced to determine whether the F1 generation was mutated. F2 embryos were obtained by mating F1 embryos with the fish with the same mutation. The F2 embryos were raised to adulthood. DNA was extracted from the zebrafsh tail, amplifed via PCR, and compared with DNA from the control group to confirm the mutation. The mutated PCR products were sequenced to verify the mutation. All animal experiments were approved by the Institutional Animal Care and Use Committee of Shanghai Ocean University (Approval number: SHOU-DW-2024-311).

### 2.2. Analysis of miR499 Knockout Efficiency

Total miRNA was isolated from 100 pooled 6-day post-fertilization (dpf) WT and miR499^−/−^ zebrafish larvae using the MiPure Cell/Tissue miRNA Kit (RC201, Vazyme, Nanjing, China). Reverse transcription was performed using the miRNA 1st Strand cDNA Synthesis Kit (MR101-01, Vazyme, China) with gene-specific stem-loop primers for miR499-5P(GTCGTATCCAGTGCAGGGTCCGAGGTATTCGCACTGGATACGACTAAACA) and miR499-3P (GTCGTATCCAGTGCAGGGTCCGAGGTATTCGCACTGGATACGACAGCACA). U6 small nuclear RNA was used as an endogenous control. Semi-quantitative RT-PCR was conducted to assess the knockout efficiency of both mature miRNA strands. Primer sequences are listed in Table 1.

### 2.3. RNA Sequencing

To explore the candidate pathway and possible mechanism of IB development regulated under miR499, total RNA was extracted from muscle with IBs using a RNAiso Plus Extraction Kit (9108, Takara, Kusatsu, Japan) according to the manufacturer’s instruction. Three fish were selected randomly from WT and miR499^−/−^ mutant line and were mixed for deep sequencing analysis. Libraries were sequenced on an Illumina Nova-Seq 150 bp PE run by Novogene (Novogene, Beijing, China). The quality of the sequencing output was assessed using FastQC v.0.11.7. The clean reads were aligned to the *Danio rerio* reference genome (Accession number: GCA_000002035_4) using Hisat2 (v2.0.5) with default parameters. The abundances of transcripts were quantified and normalized based on fragments per kilo bases per million reads (FPKM). Genes differentially expressed between the running water group and the control group were analyzed using DESEq2 (v1.20.0). Genes with qvalue (Adjusted *p* value) < 0.05, |log2FoldChange| > 1 were identified as significantly differentially expressed genes (DEGs). Kyoto Encyclopedia of Genes and Genomes (KEGG) analysis was performed to identify significantly enriched pathways associated with DEGs. The clusterProfiler package in R was used to test the statistical enrichment of DEGs in KEGG pathways. Selected DEGs were validated by RT-qPCR.

### 2.4. Bone Staining and Morphometric Analysis

For adult zebrafish (over 3 months old): Intermuscular bones (IBs) were visualized using Alizarin Red S staining as previously described [34] with minor modifications. Briefly, fish were fixed in 4% paraformaldehyde (PFA) for 24 h, followed by digestion in 1% trypsin (*w*/*v*) and clearing in 1% potassium hydroxide (KOH, *w*/*v*). After rinsing, body pigments were bleached using an equal-volume mixture of 3% hydrogen peroxide (H_2_O_2_) and 0.5% KOH until complete decolorization. Samples were then stained with 2 mg/mL Alizarin Red S (dissolved in 1% KOH) and gradually transferred to a fresh solution of 50% glycerol containing 0.1% KOH for preservation. Stained specimens were imaged under a dissecting microscope (SMZ1500, Nikon, Tokyo, Japan). Individual IBs were isolated from stained fish under the dissecting microscope, sequentially arranged on glass slides, and photographed. The sequential IB images were spliced into complete IB sequences using the Adobe Photoshop software. The number and ossified area of IBs were quantified from the digital images using *ImageJ* software (ImageJ v1.47, NIH, Bethesda, ML, USA).

For larval zebrafish (20–40 days post-fertilization, dpf): Wild-type (WT) and miR499 knockout (miR499^−/−^) mutant zebrafish were separately mated, and fertilized embryos were incubated under standard conditions. Larvae with normal development (no morphological abnormalities) were selected under a dissecting microscope and reared in 1L glass tanks (50 larvae per tank) to minimize environmental variation. All tanks were maintained with consistent feeding regimes (e.g., a daily ration of brine shrimp nauplii) and photoperiod (14 h light:10 h dark). To track the initial emergence of IBs, three larvae from each group (WT and miR499^−/−^) were sampled daily once approaching the typical IB emergence window. Upon the first detection of IBs, 20 larvae were randomly selected from each group for bone staining (as described above) to quantify the number of emerged IBs. The emergence rate of IBs was evaluated by comparing the number of IBs in synchronously hatched larvae at the initial emergence stage.

For analysis of IB growth rate: Zebrafish at 30, 60, 90, 120, and 180 dpf were sampled (three individuals per stage). Samples were fixed in 4% PFA, subjected to bone staining and clearing as described above, and imaged under a microscope. To quantify IB growth, the ossified length of epineural bones (dorsal to the vertebral axis) and epipleural bones (ventral to the vertebral axis) at the seventh vertebra was measured using ImageJ (v1.47). To account for individual differences in body size, IB length was normalized to body length (BL). The relative length of IBs was calculated as: Relative length = Length of epineural/epipleural bones/Body length (BL). Growth rate curves of IBs were plotted based on these relative lengths across developmental stages.

### 2.5. Skeletal Muscle Fiber Analysis

For the muscle histological analysis, three-month-old zebrafish from the miR499^−/−^ and WT groups were anesthetized with 200 mg/L of tricaine methanesulfonate solution. Subsequently, all samples were cut off all fins and scales and fixed in 4% PFA at 4 °C for 24 h. Transverse sections (7 μm thickness) were prepared from paraffin-embedded trunk muscle of three-month-old zebrafish. For immunofluorescent staining according to previous protocols [45], sections were incubated with the F59 antibody (F59, DSHB, USA), an anti-myosin heavy chain antibody specific to slow muscle fibers, followed by an Cy3-labeled Goat Anti-Mouse IgG (H+L)) secondary antibody. The sections were imaged using a Leica TCS SP8 laser scanning confocal microscope (TCS SP8, Leica, Wetzlar, Germany). For histological analysis, sections were stained with hematoxylin and eosin (H&E). The sections were imaged using a light microscope (LeicaDM IL LED, Leica, Germany). The cross-sectional area (CSA) of slow muscle and the number of fast muscle fibers were quantified using ImageJ (ImageJ v1.47, NIH, USA).

### 2.6. Locomotion Behavior Analysis

Swimming performance was assessed as previously described [34]. Ten fish from each strain were collected at two distinct time points: 30 dpf, defined by the initial emergence of IBs, and 3 mpf, when IB ossification is fully accomplished. To characterize zebrafish locomotion behavior, swimming kinematics were captured using a high-speed camera and analyzed based on three parameters: tail-beat amplitude (A, mm), tail-beat frequency (Tf, Hz: 1/s), and stride length (SL, mm). Tail-beat frequency (Tf) was calculated as the reciprocal of the duration required for the tail to complete one full beat cycle. The stride length (distance covered during one tail beat) and body wave length (distance covered during one body wave) were also determined. Throughout the remainder of the text, all lengths are normalized by standard body length (BL); hence, tail beat amplitude and stride length are expressed as specific values. Prior to testing, fish were acclimated to the test tank for 15 min to minimize stress-induced behavioral artifacts. The temperature of the testing room was maintained at 27–28 °C throughout the experiment. During video recording, each fish was individually transferred into a plastic tank (25 length × 25 width × 10 height cm) containing chlorine-free bore-well water. The behavior of each fish was captured with a MD4256 digital camera (Nikon), attached to a high-speed camera system (IDT Y3-S2 Editon, Integrated Design Tools Inc., La California, Italy) running on a PC. Data were acquired at 1000 frames/s, and 100 swimming sequences were recorded for each zebrafish line. All tests were performed using three-month-old zebrafish. Animals were first kept to the testing tank. The temperature in the testing room was kept at 27~28 °C.

### 2.7. X-Ray Microtomography (Micro-CT)

Three-month-old zebrafish were digested with 1% trypsin to transparency prior to scanning. Samples were scanned using an NSI X50 micro-CT system (North Star Imaging) at 60 kV and 280 μA, with an isotropic voxel size of 2.88 μm. Three-dimensional reconstructions were generated using the efX-CT software (North Star Imaging, Inc., Rogers, MN, USA). By adjusting the gray value threshold, the image of bones remained, while the soft tissues were virtually invisible.

### 2.8. Real Time Quantitative PCR (RT-qPCR)

At 30 dpf, when intermuscular bones initially emerge, and at 3 months post-fertilization (mpf), when IB development is complete, muscle tissues containing IBs were collected from zebrafish. For adult zebrafish at 3 mpf, 10 individuals from each of the two strains were randomly selected. IBs were carefully dissected under a dissecting microscope using ultrafine-tipped forceps, with minimal residual muscle tissue attached to the bone surface. Approximately 30–50 IBs were collected per individual. Given the small size of individual IBs, to ensure sufficient RNA, IBs from all 10 individuals (per strain) were pooled for subsequent RNA extraction. Total messenger RNA was extracted by the RNAZol^®^RT (Invitrogen, Waltham, MA, USA) and quantified with NanoDrop (Thermo Scientific, Waltham, MA, USA). A RevertAid first cDNA synthesis kit (K1622, Fermentas, Waltham, MA, USA) was used to synthesize first-strand cDNA from total zebrafish RNA according to the manufacturer’s instructions. qRT-PCR was performed using iQ SYBR Green Supermix (Bio-Rad Laboratories, Hercules, CA, USA) on a Bio-Rad iCycler using elfa as control, and data were analyzed using the 2^−ΔΔCt^ method. The 20 μL mixture of PCR consisted of 10 μL Hieff^®^ qPCR SYBR Green Master Mix (No Rox), 0.4 μL forward primer (10 μM), 0.4 μL reverse primer (10 μM), and 2 μL DNA template, and RNase-free water was added to it. The qPCR conditions were as follows: denaturation at 95 °C for 5 min, 40 cycles of denaturation at 95 °C for 10 s, annealing at 60 °C for 20 s, and extension at 72 °C for 20 s. The primer sequences used to perform quantitative real-time PCR are listed in Table 1. Each sample was analyzed in triplicate.

### 2.9. Statistical Analysis

All data were analyzed by two-sided unpaired *t*-test using the statistical package for GraphPad Prism9.0. The error bars represent the SD. The *p* values were calculated and are indicated in the figure legends. ns *p* > 0.05; * *p* < 0.05; ** *p* < 0.01; *** *p* < 0.001.

## 3. Results

### 3.1. Construction of miR499 Zebrafish Mutant by CRISPR/Cas9 Technology

**Figure 1:**
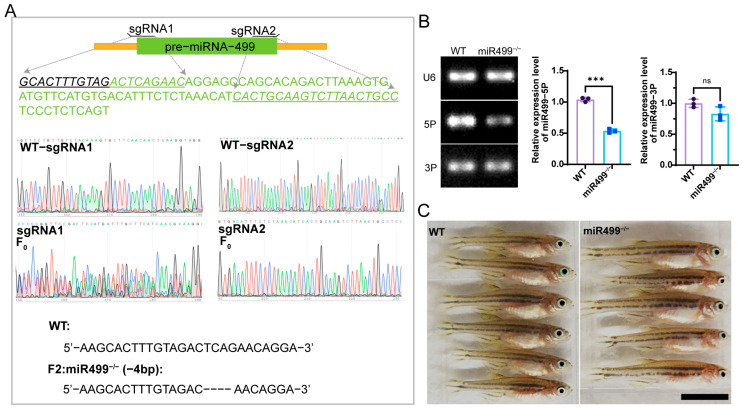
Generation and characterization of miR499 knockout. (**A**) Schematic illustration of miR499 mutation induction by the CRISPR/Cas9 system with two sgRNAs, and sequencing results of WT and miR499 mutant (F2) with 4—bp deletion (named miR499^−/−^). Underlined and italicized text indicates the sgRNA target sequence. Green font denotes the pre-miRNA-499 sequence. (**B**) Semi-quantitative RT-PCR analysis of dre—miR499—5P and dre—miR499—3P expression in WT and miR499^−/−^ fish, with U6 as a loading control. *** *p* < 0.001. ns: no significance; (**C**) Representative images of WT and miR499^−/−^ fish at 30 days post-fertilization. Scale bars = 50 mm.

To investigate the role of muscle in the development of IBs, we first generated miR499 knockout involving muscle fiber function using the CRISPR/Cas9 technology. As shown in Figure 1A, two single-guide RNAs (sgRNA1 and sgRNA2) were designed to target the miR499 genomic locus (GRCz11, chr11: 26, 541, 321–26, 541, 420). The sequencing results of WT- and F_0_-generation zebrafish injected with sgRNA1 or sgRNA2 revealed distinct peak patterns, confirming successful mutagenesis at the sgRNA1 target site. By crossing the effectively edited F_0_ zebrafish with WT fish and subsequently performing self-crossing, we successfully obtained a mutant line (F2) carrying a 4 bp deletion, designated as miR499^−/−^ (Figure 1A). To assess the effect of this mutation on miR499 expression, semi-quantitative RT-PCR was conducted to detect the 5P and 3P fragments of miR499-derived small RNAs and its target gene *myh7ba*. As shown in Figure 1B, the transcript level of dre-miR499-5P was significantly reduced by 50% in miR499^−/−^ zebrafish compared to WT, whereas the expression of dre-miR499-3P was reduced but without a statistically significant difference (The original Gel image for this figure can be found in Figure S1). The expression level of *myh7ba* mRNA is also like miR499-3P (The original Gel image for this figure can be found in Figure S2). We further examined phenotypic differences between miR499 knockout and WT during development. The miR499 mutant were viable and fertile, with no overt developmental abnormalities. Figure 1C compares the overall morphology of WT and miR499^−/−^ fish at 30 days post-fertilization. Apart from a slight reduction in body size, no other obvious morphological differences were observed between the two groups.

### 3.2. Regulation of sox6 and Myosin Heavy Chain-Related Genes by miR499 Knockout

**Figure 2:**
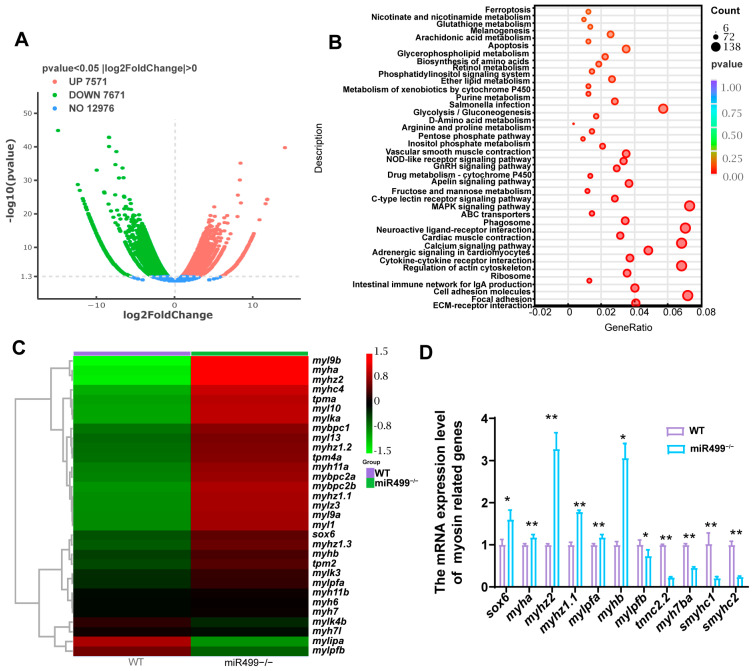
Transcriptomic analysis of muscle tissue in WT and miR499^−/−^ zebrafish at 3 months old. (**A**) Volcano plot showing differentially expressed genes between WT and miR499^−/−^ zebrafish (red dots: up—regulated genes; green dots: down—regulated genes; blue dots: genes with no significant change); (**B**) Bubble plot of pathway enrichment analysis for differentially expressed genes (Top40). The size of bubbles represents gene count, and color represents *p*–value. Red indicates high expression. Green indicates low expression; (**C**) Hierarchical clustering of the muscle-related DEGs in miR499^−/−^ mutants compared with WT; (**D**) qPCR validation of expression levels of selected myosin—related and red muscle—specific genes. ** *p* < 0.01, * p < 0.05.

The RNA sequencing of muscle tissue from miR499 knockout zebrafish identified 15,242 significantly differentially expressed genes (DEGs), comprising 7571 upregulated and 7671 downregulated genes (Figure 2A). Notably, among these DEGs, we identified 60 genes closely associated with muscle development and function, with 49 being significantly upregulated and 11 downregulated (Supplementary Table S1). The expression of *sox6*, a transcriptional regulator known to specify muscle fiber type, was found to be significantly upregulated (*p* = 8.611 × 10^−5^, log2(FC) = 1.754). KEGG pathway enrichment analysis showed that, among the top 40 enriched pathways (based on corrected *p*-value) for the WT vs. miR499^−/−^ up-regulated genes (Figure 2B), including pathways involved in muscle contraction and signaling, such as Cardiac muscle contraction, Vascular smooth muscle contraction, and the Calcium signaling pathway. Pathways related to energy metabolism (Glycolysis/Gluconeogenesis, Fructose and mannose metabolism) and extracellular matrix and adhesion (ECM-receptor interaction, Focal adhesion, Cell adhesion molecules) were also significantly enriched. The MAPK signaling pathway was also notably enriched. Moreover, these pathways involve genes with decreased expression after miR499 loss. Major focuses include carbohydrate metabolism (e.g., glycogenolysis, starch/sucrose metabolism, glycolysis/gluconeogenesis), protein processing (e.g., RNA degradation, protein processing in endoplasmic reticulum), intracellular transport (e.g., nucleocytoplasmic transport, vesicle-mediated transport), and cell cycle/division (e.g., cell cycle, DNA replication). This indicates that miR499 loss impairs energy metabolism, protein homeostasis, cellular trafficking, and cell cycle progression. The hierarchical clustering of the muscle-related DEGs showed that multiple myosin heavy chain (MyHC) isoforms, including those characteristics of both fast-twitch (e.g., *myh6*, *myha*, *myhz2*) and slow-twitch/cardiac muscle (e.g., *myh7*, *myh7l*, *myh11a*, *myh11b*), were significantly downregulated. The key regulatory components of the sarcomere, such as myosin light chains (e.g., *mylpfa*, *myl9a/b*), tropomyosins (e.g., *tpma*, *tpm4a*, *tpm2*), and myosin-binding protein C (*mybpc1*, *mybpc2a/b*), were also upregulated. To validate the transcriptomic findings and some key gene-related slow-twitch muscle, qPCR was performed on a subset of genes (Figure 2D). The results validated the significant upregulation of *sox6* (*p* < 0.05) and fast myosin heavy chain genes *myha*, *myhz2*, *myhz1.1*, and *myhb*. Conversely, the expression of *mylpfb*, *tnnc2.2*, *myh7ba*, and the slow muscle-specific genes *smyhc1* and *smyhc2* was significantly downregulated (*p* < 0.01).

### 3.3. Effects of miR499 Knockout on Zebrafish Muscle

**Figure 3:**
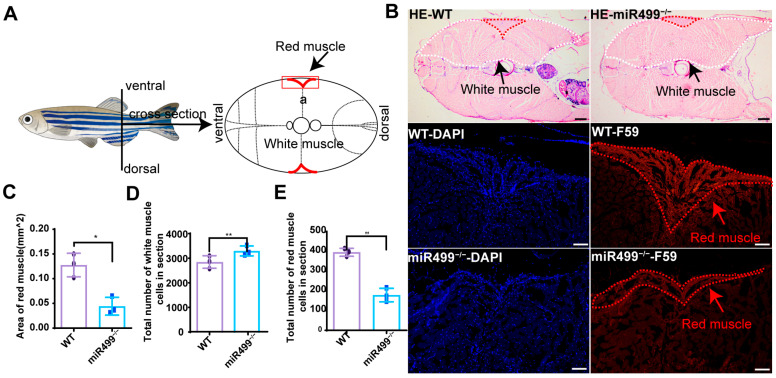
Histological analysis of muscle in WT and miR499^−/−^ zebrafish at 3 months old. (**A**) Diagrams of body areas of zebrafish used for histological analysis. a represents red muscle; (**B**) Tissue sections and immunofluorescent staining by HE staining and F59 antibody separately in zebrafish muscle. Red arrows indicate red muscle. White dotted lines outline white muscle areas, and black arrows indicate white muscle. Red dotted lines outline red muscle areas, and red arrows indicate white muscle. Scale bar: 200 µm; (**C**) Quantification of red muscle area. *N* = 3. * *p* < 0.05; (**D**) Quantification of total number of white muscle cells in section. *N* = 3. ** *p* < 0.01. (**E**) Quantification of total number of red muscle cell. *N* = 3. ** *p* < 0.01.

Given that *sox6* is implicated in red and white muscle development and miR499 mutation leads to *sox6* upregulation, we performed histological analysis to investigate muscle changes. Figure 3A shows diagrams of body areas of zebrafish used for histological analysis. Representative micrographs of HE-stained sections are shown in Figure 3B. The cross-sectional area of the slow-twitch (red line) muscle domain, as delineated by F59 immunostaining, was significantly reduced in mutants (0.044 mm^2^) compared to wild-type controls (0.13 mm^2^) (Figure 3C). Concurrently, analysis of the HE-stained sections showed that the total number of fast-twitch (white line) muscle cells was markedly increased in miR499 knockout (3297/section) relative to wild-type fish (2846/section) (Figure 3D). In addition, we quantified the number of red muscle cells in cross-sections, as shown in Figure 3E. The average number of red muscle cells in miR499 mutant mice was 179 cells/section, which was a statistically significant reduction compared to that in wild-type (WT) mice (396 cells/section) (*p* < 0.01).

### 3.4. Impacts of miR499 Knockout on Zebrafish Locomotion Capability

**Figure 4:**
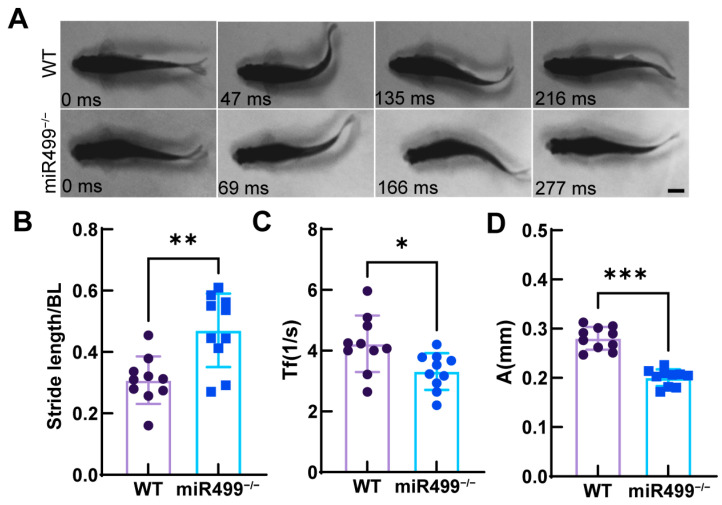
Kinematic parameters in WT zebrafish and miR499^−^/^−^ mutant at 3month old. (**A**) An example of a cruise swim during cyclic swimming. Scale bar: 5 µm (**B**–**D**) Statistical analysis of red muscle area. Comparison of A (tail beat amplitude), SL (stride length), Tf (tail beat frequency). Values for all data are means ± SD. *N* = 10. Two-tailed Student’s *t*-test were conducted, * *p* < 0.05; ** *p* < 0.01; *** *p* < 0.001.

To evaluate the effect of miR499 knockout on locomotion capability, we analyzed the swimming behavior of WT and miR499^−/−^ zebrafish. Figure 4A displays sequential images of tail movements during cyclic swimming, revealing that miR499^−/−^ zebrafish had a longer tail-beatcycle (approximately one cycle in 277 ms) compared to WT zebrafish (approximately one cycle in 216 ms). Further quantitative analyses revealed that the relative stride length (normalized to body length) was significantly higher in miR499^−/−^ zebrafish (~0.63) compared to WT fish (~0.49) (Figure 4B). Meanwhile, the tail-beat amplitude was smaller in miR499^−/−^ zebrafish (~0.20 mm) than in WT zebrafish (~0.28 mm) (Figure 4C). Additionally, the tail-beat frequency (Tf) was also significantly reduced in miR499^−/−^ zebrafish (Figure 4D). In summary, miR499^−/−^ zebrafish exhibited enhanced locomotion capability, as they could fulfill swimming demands with fewer tail beats, despite having lower tail-beat frequency and amplitude, which was likely compensated for by the increased stride length.

### 3.5. Role of miR499 in Intermuscular Bone (IB) Development in Zebrafish

**Figure 5:**
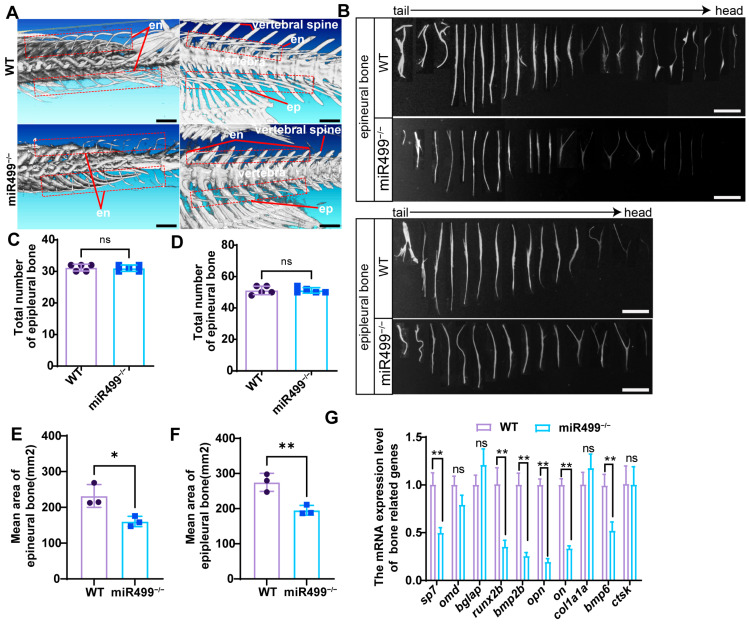
miR499 diminished the ossification of intermuscular bones in zebrafish. (**A**) Micro-CT imaging of whole-body skeletons, revealing reduced IB ossification area in miR499^−/−^ zebrafish. en: epineural bone; ep: epipleural bone. Red dashed boxes indicate partial intermuscular bones (IBs); (**B**) Isolated IBs from zebrafish (scale bar = 500 μm); (**C**,**D**) Quantification of the number of epineural bones and epipleural bones. *N* = 5; (**E**,**F**) Quantification of IB ossification mean area at 3 mpf. *N* = 3. * *p* < 0.05, ** *p* < 0.01; (**G**) qRT-PCR analysis of bone-related gene expression in IB tissues separated from muscle. Data are presented as mean ± SD; * *p* < 0.05, ** *p* < 0.01. ns: no significance.

To elucidate the function of miR499 in bone development, we first employed micro-computed tomography (Micro-CT) to visualize the whole-body skeletal architecture of WT and miR499^−/−^ zebrafish. As shown in Figure 5A, the ossification of IBs was notably impaired in miR499^−/−^ zebrafish compared with WT, whereas other skeletal components showed no obvious abnormalities. Subsequently, we dissected IBs from zebrafish to analyze their ossification features; the isolated IBs are presented in Figure 5B. Quantifying the number of calcified IBs revealed no significant difference in the total number of IBs (including epineural and epipleural bones) between miR499^−/−^ and WT zebrafish (Figure 5C,D), with the average number of epineural bones being 51 ± 1 and epipleural bones being 31 ± 1 in both genotypes. However, the morphology of IBs in miR499^−/−^ zebrafish was simpler, restricted to types such as I-shape, Y-shape (Figure 5A,C,D). For the ossification area, statistical analysis confirmed that the ossification area of IBs was significantly reduced in miR499^−/−^ zebrafish relative to WT (Figure 5E,F). Specifically, the mean ossification area of epineural bones in miR499^−/−^ zebrafish decreased from 231.8 ± 32 mm^2^ (in WT) to 160.4 ± 14 mm^2^, and the mean ossification area of epipleural bones decreased by from 274.9 ± 25 mm^2^ (in WT) to 195 ± 14 mm^2^. The total ossification area decreased by 27%. Moreover, we performed qRT-PCR to detect the expression of bone-related genes in adult IB tissues. As depicted in Figure 5G, the expression levels of *sp7*, *runx2b*, *bmp2b*, *opn*, *on*, and *bmp6* were significantly downregulated in miR499^−/−^ zebrafish compared with WT. In contrast, the expression of *bglap* and *col1a1a* tended to increase but without statistical significance.

### 3.6. Regulatory Function of miR499 in Intermuscular Bone (IB) Formation

**Figure 6:**
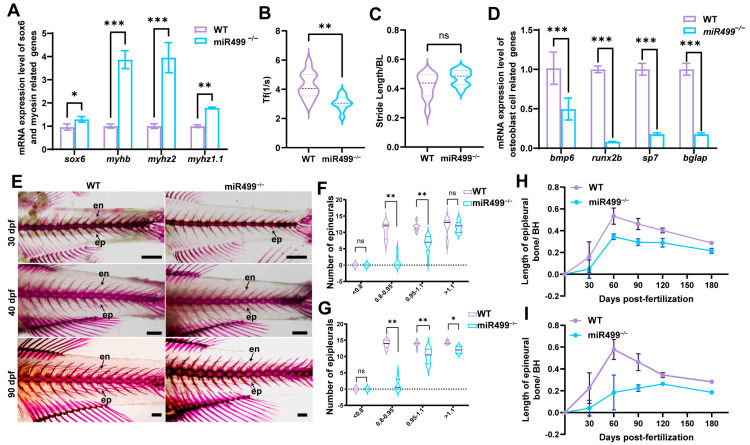
miR499 deficiency delays IBs ossification and modulates muscle gene expression and locomotion. (**A**) qPCR analysis of sox6 and myosin-related genes in muscle tissues of WT and miR499^−/−^ zebrafish at 30 dpf. (**B**,**C**) Tail-beat frequency and (**C**) stride length of WT and miR499^−/−^ zebrafish at 30 dpf. Tf: Tail-beat frequency, Stride length was normalized by Body Length. (**D**) qPCR analysis of osteogenic genes in muscle tissues of WT and miR499^−/−^ zebrafish at 30 dpf. (**E**) Alizarin red staining of IBs in WT and miR499^−/−^ zebrafish at 30, 40, and 90 dpf. Scale bars = 1 mm. Arrows represent IBs. en: epineural bone; ep: epipleural bone. (**F**,**G**) Quantification of epineural bone (**F**) and epipleural bone (**G**) in WT and miR499^−/−^ zebrafish across different body length ranges at 30 dpf. (**H**,**I**) Ossification rate of two IBs epineural bone and epipleural bone in miR499^−/−^ mutant and WT. The relative length of IBs was calculated as: Relative length = Length of (epineural/epipleural bones)/Body length (BL). Growth rate curves of IBs were plotted based on these relative lengths across developmental stages. *N* = 3. * *p* < 0.05, ** *p* < 0.01, *** *p* < 0.001. ns: no significance.

To investigate the role of miR499 in IB development, we performed continuous tracking of IB formation in zebrafish. In WT larvae, IBs initiated in the tail region at 30 dpf. qPCR analysis of muscle tissue at this stage showed that the mRNA levels of *sox6* and representative myosin-related genes (*myhb*, *myhz2*, *myhz1.1*) were significantly upregulated in miR499^−/−^ mutants compared to WT controls (Figure 6A). At 30 dpf, the tail-beat frequency of miR499^−/−^ larvae was significantly reduced, while stride length showed a slight but non-significant increase (Figure 6B,C). In addition, the expression of osteogenic genes (*bmp6*, *runx2b*, *sp7*) was downregulated in mutants (Figure 6D). We further quantified calcified IBs at 30 dpf and observed a notable reduction in miR499^−/−^ zebrafish, particularly among individuals with a body length of 0.8~0.95 cm, where the difference was most evident (Figure 6E–G). As development continued, IBs appeared in all myosepta of mutant fish, and the number of ossified IBs eventually reached levels comparable to those in WT. To assess the dynamics of IB ossification, we monitored the growth rate of IBs at the 7th vertebra from 30 to 180 dpf. The IB growth rate in both genotypes exhibited a biphasic pattern, rising initially before declining with developmental progression. Throughout this period, however, the ossification rate in miR499^−/−^ mutants was consistently and markedly slower than in WT (Figure 6H,I). Collectively, these results demonstrate that miR499 deficiency delays the onset and slows the ossification of IBs, concurrent with altered muscle gene expression and locomotor behavior during early development.

## 4. Discussion

**Figure 7:**
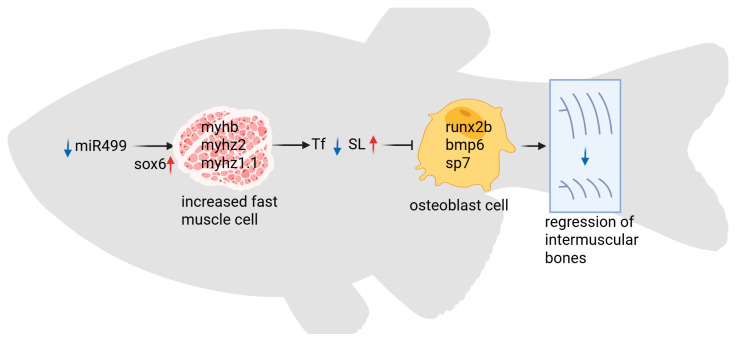
Simplified model of intermuscular bones regulated by miR499 in zebrafish. Tf: Tail beat frequency; SL: stride length.

In this study, we systematically investigated the role of muscle-derived miR499 in regulating IB development and muscle fiber type specification. Our investigation demonstrates that knocking out the miR499 results in a marked upregulation of the transcription factor *sox6* in skeletal muscle, thereby inducing myosin heavy chain gene expression profiles. The mutation of miR499 upregulated the expression of fast-twitch muscle fiber genes (including *myhz2* and *myha*) and downregulated slow-twitch fiber genes (such as *smyhc1* and *smyhc2*). These transcriptomic alterations were subsequently validated at the histological level, revealing a significant increase in fast-twitch muscle fiber number and corresponding reduction in slow-twitch muscle cross-sectional area in mutants. Functionally, this fiber-type transition may mediate distinct modifications in swimming kinematics, manifested as decreased tail-beat frequency with increased stride length. Most notably, the mutation of miR499 may directly impair IB development, evidenced by the delayed emergence, reduced ossification rate, and simplified morphological complexity of IBs. These morphological abnormalities were associated with a significantly suppressed expression of core osteogenic regulators, including *runx2b* and *bmp6* (Figure 7).

Existing studies have clearly established that miR499 is a key regulator of vertebrate skeletal muscle fiber type switching and its mechanism of action—primarily through targeting *sox6*—is highly conserved across species [39,41,42,46]. Sox6 has been proposed to play a conserved role in vertebrate skeletal muscle fiber type specification [39]. In zebrafish, *sox6* is a direct target gene of the miR499 family, and miR499 forms a regulatory loop with Prdm1a [39]. Specifically, miR499 inhibits *sox6* mRNA expression at the translational level, confining Sox6 to the fast-twitch muscle lineage, thereby maintaining the differentiation fate of slow-twitch muscle fibers [39]. This mechanism is equally conserved in terrestrial vertebrates: in chickens, miR499 expression is significantly higher in slow-twitch muscle than in fast-twitch muscle, and dual-luciferase reporter assays have confirmed that miR499 directly targets and inhibits *sox6* [47]. In porcine skeletal muscle, miR499-5p not only validates *sox6* as a target gene but also synergistically promotes the expression of slow-twitch muscle genes [48]; notably, active immunization against Sox6 protein can even improve pig quality [49]. In Nile tilapia (*Oreochromis niloticus*), miR499 is highly expressed in slow-twitch muscle, showing a negative correlation with the low expression of sox6, and regulates its target gene through a dual mechanism of mRNA instability and protein translation attenuation [46]. Consistent with this evolutionarily conserved regulatory framework, our study demonstrated that the miR499 mutant in zebrafish caused a significant upregulation of the transcriptional repressor Sox6, which in turn led to a marked reduction in red muscle cell number (179 cells/section in mutants vs. 396 cells/section in WT) and a concomitant increase in white muscle cells. Furthermore, we observed alterations in the expression profile of myosin heavy chain (MyHC) genes, characterized by the upregulation of fast-contracting muscle genes (e.g., *myhz2*, *myha*) and the downregulation of slow-contracting muscle genes (e.g., *smyhc1*, *smyhc2*). These molecular shifts were corroborated at the histological level, where we observed an increase in fast-twitch muscle cell number concomitant with a reduction in slow-twitch muscle cross-sectional area. Collectively, these results not only reinforce the evolutionary conservation of the miR499-Sox6 axis in regulating muscle fiber type specification but also provide in vivo evidence for its critical role in shaping the cellular composition and phenotypic characteristics of muscle tissue in zebrafish.

Beyond its regulation of *sox6*, miR499 participates in multiple other biological processes and targets that underpin muscle physiology. Moreover, miR499 exerts its multifaceted roles in vertebrate muscle development via the post-transcriptional regulation of a suite of target genes beyond Sox6, including PTEN, PDCD4, Fnip1, and TGFβR1, which are involved in muscle fiber type specification, cell survival, and mitochondrial dynamics [50,51,52,53,54]. In our study, while we confirmed that miR499 mutation upregulates sox6, it is important to acknowledge the limitations of our work in the context of miR499’s pleiotropic functions. Specifically, although RNAseq identified expression correlations for genes like *fnip1*, *ptenb*, and *tgfbrap1*, it does not establish direct targeting relationships. Thus, the candidacy of these genes as direct miR499 targets in zebrafish requires validation through rigorous approaches such as luciferase reporter assays. Furthermore, the precise mechanisms underlying the dysregulation of *ptenb* and *tgfbrap1* remain unclear, necessitating future investigations to distinguish direct miR499-mediated effects from secondary transcriptional consequences. These limitations highlight critical avenues for future research to fully delineate the complex regulatory network of miR499 in zebrafish muscle development and fiber type specification.

Notably, the molecular and histological alterations in muscle fiber composition observed in miR499 mutants directly translated to distinct changes in swimming locomotion: tail-beat frequency (TBF) decreased, while stride length (SL) increased. Zebrafish exhibit a canonical carangiform swimming mode, characterized by undulatory waves confined to the posterior third of the body, a streamlined morphology, and precise coordination between myotomal muscle function and propulsive efficiency [55]. Fast-twitch muscle (white muscle) has significantly higher myosin ATPase activity than slow-twitch muscle (red muscle), generating greater tension during contraction (approximately 1.2-fold that of red muscle). This property enables it to drive larger amplitude undulations of the posterior trunk in carangiform swimmers [56]. Increased trunk bending displaces a larger volume of water, creating stronger propulsive counterforces and thereby extending the distance traveled per tail beat (stride length, SL). Thus, the increased proportion of white muscle in miR499 mutants directly contributes to the observed increase in SL. In contrast, red muscle is rich in mitochondria and sustains ATP production via oxidative metabolism, providing energy for high-frequency tail beats (TBF) during steady swimming in carangiform species—a process that requires repeated muscle fiber activation and substantial energy consumption [57]. miR499 mutation reduces the proportion of red muscle, directly impairing oxidative metabolic capacity. This failure to meet the energy demands of high-frequency contractions ultimately leads to a decrease in TBF [53].

IBs—specialized membranous bones within the myosepta of cyprinid fish—have recently become a research focus, with studies centering on deciphering their developmental mechanisms and improving aquaculture-related traits. However, research on the regulatory roles of muscle and locomotion in IB development is still in its preliminary stages, and the involvement of non-coding RNAs (including miRNAs) in this process remains largely uncharacterized. Existing studies have confirmed that IB development relies on the precise regulation of osteogenesis-related coding genes, and some of these genes have potential associations with muscle function. For example, the knockout of the *runx2b* gene in zebrafish leads to the complete absence of IBs, while deletion of *bmp6* gene causes a partial or complete loss of IBs—confirming these two genes as key regulators of IB formation [16,17]. In zebrafish with *asb15* knockout, IB number is significantly reduced by 30–40%, accompanied by an altered expression of bone development-related genes such as *sox6* and *bmp6*; notably, *asb15* itself is involved in skeletal muscle development and metabolism, suggesting that muscle-related genes may indirectly regulate IB development [58]. Regarding developmental regulatory mechanisms, the ossification order of IBs is closely associated with fish locomotion patterns: in fish that rely on tail oscillation for movement (e.g., *D. rerio*, *H*. *molitrix*), IBs ossify from the posterior to the anterior; in contrast, in fish that depend on head oscillation (e.g., eels), the ossification order of IBs is reversed [30,34]. This observation implies that mechanical stress generated by locomotion may be involved in regulating IB development. Our study is the first to link miR499 to IB development, showing that miR499 knockout delays IB ossification in zebrafish: at 30 dpf, the number of calcified IBs decreased, ossification area was reduced, and IB morphology was simplified; concurrently, the expression of core osteogenic genes (e.g., *sp7*, *runx2b*, *bmp2b*) was downregulated. Importantly, we confirmed that miR499 only regulates the rate of IB ossification, not the final number of IBs—indicating that miR499 acts as a “temporal regulator” of IB development rather than a determinant of IB initiation. Integrating these findings with existing research, we propose a clear regulatory pathway underlying this phenomenon: First, indirect regulation via the muscle microenvironment. Existing studies have confirmed that muscle can influence bone development through paracrine signaling [47,48]. In our study, the dominance of fast-twitch muscle fibers caused by miR499 deficiency may alter the muscle secretory profile, reducing the secretion of osteogenesis-promoting factors (e.g., Bmps) or increasing the production of bone formation inhibitors. This shift would downregulate the expression of genes such as *runx2b* and *sp7* in IB-forming cells, thereby delaying IB ossification. This mechanism is consistent with the observation that altered *sox6* expression affects IB development in *asb15*-knockout zebrafish, further supporting the role of muscle-related molecules in IB regulation [58]. Second, regulatory effects mediated by locomotor mechanical stress. In our study, the reduced tail-beat frequency in miR499 mutant zebrafish inevitably decreases the periodic mechanical stimulation of the myoseptal region, where IBs develop.

## 5. Conclusions

In conclusion, building on previous research on the role of miR499 in muscle regulation, our study is the first to extend the function of miR499 to IB development in zebrafish. This work not only echoes existing hypotheses about the associations between muscle, locomotion, and bone development but also adds a critical non-coding RNA node to the molecular regulatory network of IBs. Moreover, our findings provide a novel theoretical target for improving IB-related traits in aquaculture—addressing a major challenge in the consumption and processing of cyprinid fish.

## Figures and Tables

**Table 1:** Primers used for RT-qPCR.

Symbol	Forward Primer	Reverse Primer
*miR499a-5P*	GCGCGTTAAGACTTGCAGTGA	AGTGCAGGGTCCGAGGTATT
*miR499a-3P*	CGCGCGAACATCACTTTAAGTC	AGTGCAGGGTCCGAGGTATT
*U6*	TGCTCGCTACGGTGGCACA	AAAACAGCAATATGGAGCGC
*sox6*	AGTCACTTCCTTCGCTTCCG	CCAGGTTGGCCTGTACACAT
*myha*	CAAAAAGGCAAGGCTGAGGC	TTGAAGTGTCCTCAACGACGG
*myhb*	GAGTGGGGACCCAGAAATGG	TGCCACAGAGAGTTTGCACT
*myhz2*	ATCTGGTCTCAAGGAACGCA	TTGGGAGGATTCATTGGGTGG
*myhz1.1*	GACCTGAAACTGGCCCAAGA	CAGCTCGTTCAGCCTCGATT
*mylpfb*	GGATGTGCTGGCAACAATGG	GCGCCCTTTAGCTTTTCACC
*mylpfa*	GCAGAGCCAGATTCAGGAGTAC	GTGAAGTTGATTGGGCCGCT
*tnnc2.2*	CGAGGAGGTCGATGAAGACG	CTGTCGATGTAGCCATCACCG
*myh7ba*	GACCAACAGGGAAACTGGCC	CGTTGTCACTCCTTGGGAGC
*smyhc1*	CCTGGTGTCTCAGTTGACCA	TGTGCCAGGGCATTCTTT
*smyhc2*	GACCGTCACTGTGAAGGAGG	CAGCCACTTGTAGGGGTTGAC
*bmp6*	GCTGGAATCTCGCAGGTTGT	CCATCACGGCCTACTAACCC
*runx2b*	CGGCTCCTACCAGTTCTCCA	CCATCTCCCTCCACTCCTCC
*cola1a1a*	CTGTGCCAATCCCATCATTTC	ATATCGCCTGGTTCTCCTTTC
*osteonectin*	ACTAACAACAAGACCTAC	TCCGATGTAATCTATGTG
*osteopontin*	GCCTCCATCATCATCGTA	AATCACCAAGCACCAGTA
*bmp4*	TTGTGCTGTGCATGTTTGAA	GGTCGCTTGGCTATGTGTTT
*sp7*	GGCTATGCTAACTGCGACCTG	GCTTTCATTGCGTCCGTTTT
*bglap*	GTTTGTGAAGCGTGACGTGG	ATAGGCGGCGATGATTCCAG
*omd*	ACTTGGCCTCCGAGAGAGAT	GCGAAGGGATAAGACGAGGG
*bmp2b*	CGAGATCGACCGACGGAAAT	GACCACTGCCGATTTGCTTG
*ctsk*	CTATAAAGAGATTCCTCAGGG	ACACGGGTCCCACATTGG
*eef1a1l1*	CTTCTCAGGCTGACTGTGC	CCGCTAGCATTACCCTCC

## Data Availability

The data that support the findings of this study are available from the https://figshare.com/account/articles/30355228 (accessed on 14 October 2025) or the corresponding author upon reasonable request.

## Supplementary Materials

The following supporting information can be downloaded at: https://www.mdpi.com/article/10.3390/biology14121670/s1, Figure S1: Gel images of RT-PCR in miR499 3p, 5p and *myh7ba*; Figure S2: Gel images of myh7ba in WT and miR499^−/−^ at 7dpf; Table S1: Differentially expressed genes (DEGs) in the muscle transcriptome between WT and miR499^−/−^.
