# Supplementary material for: The Mutation of myomiR miR499 Impacts the Intermuscular Bones in Zebrafish
Source: Biology (Basel). 2025 Nov 25;14(12):1670. doi: 10.3390/biology14121670 (PMC12729833; doi:10.3390/biology14121670)
Supplement: Supplementary file 1 [file biology-14-01670-s001.zip › biology-3958265-Supplementary Figures.docx]

U6


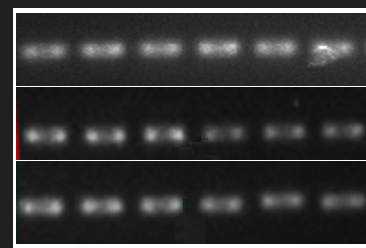


5P

3P

WT

mir499-/-

Figure S1. Gel images of RT-PCR in miR499 3p, 5p and *myh7ba*.


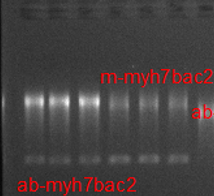


Figure S2. Gel images of myh7ba in WT and mir499-/- at 7dpf.
